# Clinical evaluation of a matrix metalloproteinase-12 cleaved fragment of titin as a cardiovascular serological biomarker

**DOI:** 10.1186/1479-5876-10-140

**Published:** 2012-07-06

**Authors:** Efstathios Vassiliadis, Lars M Rasmussen, Inger Byrjalsen, Dorthe Vang Larsen, Rajiv Chaturvedi, Susanne Hosbond, Lotte Saabye, Axel CP Diederichsen, Federica Genovese, Kevin L Duffin, Qinlong Zheng, Xiaoliang Chen, Diana J Leeming, Claus Christiansen, Morten A Karsdal

**Affiliations:** 1 Nordic Bioscience A/S, Herlev Hovedgade 207, DK-2730, Herlev, Denmark; 2 School of Endocrinology, University of Southern Denmark, Odense, Denmark; 3 Department of Clinical Biochemistry and Pharmacology, Odense University Hospital, Odense, Denmark; 4 Division of Cardiology, Hospital for Sick Children, Toronto, Canada; 5 Department of Cardiology, Odense University Hospital, Odense, Denmark; 6 Eli Lilly and Company, Indianapolis, IN, USA; 7 Nordic Bioscience Beijing, Beijing, China

**Keywords:** Titin, CVD, MMP-12, Cardiovascular, Acute myocardial infarction, Biomarker, Neoepitope

## Abstract

**Background:**

Titin is a muscle-specific protein found in cardiac and skeletal muscles which is responsible for restoring passive tension. Levels and functioning of titin have been shown to be affected by cardiac damage. Due to the inherent difficulty of measuring titin levels in vivo in a clinical setting, we aimed to develop an assay that could reliably measure fragments of degraded titin in serum and potentially be used in the assessment of cardiac muscle damage.

**Methods:**

A competitive ELISA was developed to specifically measure levels of the titin sequence 12670’ NVTVEARLIK 12679’, derived by the degradation of titin by matrix metalloproteinase (MMP)-12. Serum samples from 90 individuals were divided into 3 equally sized groups. One group had been diagnosed with acute myocardial infarction (AMI) while the remaining two were asymptomatic individuals either with CT-scan signs of coronary calcium (CT-plusCa) or without coronary calcium (CT-noCa).

**Results:**

Mean geometric levels of the titin fragment in the CT-noCa group were 506.5 ng/ml (±43.88). The CT-plusCa group showed 50.6% higher levels of the marker [763 ng/ml (±90.14)] (P < 0.05). AMI patients showed 56.3% higher levels [792 ng/ml (±149)] (P < 0.05).

**Conclusions:**

The titin-12670 fragment is present in both individuals with undiagnosed and diagnosed CVD. The statistically significant increase in level of the marker in the AMI group is indicative that this neoepitope biomarker may be a useful serological marker in AMI.

## Background

Titin, also known as connectin, is a sarcomeric protein expressed in cardiac and skeletal muscle. It is the largest known protein in nature, with a molecular weight of up to 3700 kDa [1]. Its main function in the heart is to act as a long molecular spring by restoring passive tension during myocardial stretch and enhancing or terminating active force thus regulating the Frank-Starling mechanism of the heart [2-5]. Distinct passive stress differences have been recorded between cardiac and skeletal muscles [6]. Titin has two isoforms that are co-expressed in the sarcomere, the N2A which is the larger of the two and is found in both skeletal and myocardial muscle, and the N2B isoform which is smaller, stiffer and is found in cardiac muscle [1,7-12]. Due to the different stiffness in titin’s isoforms, it has been proposed that the adaptive or maladaptive ratio alteration between the two isoforms could affect its myocardial contractile properties [4,7,13-17]. Isoform modifications and ratio alterations were first described in animal models while clinical studies have also reported isoform changes during dilated cardiomyopathy (DCM), aortic stenosis (AS), diastolic heart failure (DHF) and ischemic heart disease (IHD) [16,18-21]. The main limitation of studies of titin lies in the methods for detecting and quantifying titin levels. The studies rely on invasive tissue extractions which are then analysed by methods such as immunoblotting and gel electrophoresis.

Extracellular matrix (ECM) components are degraded by a number of different proteases including matrix metalloproteinases (MMPs). MMP-degradation of proteins generates specific cleavage sites on fragments which in turn enable the development of new epitopes. Our group previously discussed neoepitopes that may have potential utility as biomarkers of unbalanced ECM remodeling in a number of different pathologies and can be measured in biological fluids such as serum, plasma and urine [22-27]. Key benefits of measuring biomarkers in body fluids are that this process is non-invasive and, because the specific neoepitopes represent a unique ‘fingerprint’ of the proteolytic cleavage of the protein, they identify the specific tissue being turned over and also detect whether the tissue is diseased or healthy. Since pathology-related cardiac remodeling is initiated before clinical onset and appearance of any symptoms [28], biomarkers that indicate abnormal remodeling could be utilised for early diagnosis of cardiovascular disease (CVD). The cardiac-specific markers troponin I and T (cTnI & cTnT) are already used to closely monitor myocardial damage and related pathological events [29-33]. Titin has been previously shown to be degraded by MMP-2 localised in the Z-disk of the cardiac sarcomere [34,35]. However to our knowledge no biomarkers based on titin-relevant neoepitopes resulting from the degradation activity of either MMP-2 or other metalloproteinases have been described.

During digestion of human tissue by an array of exogenous metalloproteinases, a large number of proteolyzed peptides have been identified using mass spectrometry [36]. Among these, a titin specific fragment 12670’ NVTVEARLIK 12679’ was identified to have been cleaved specifically by MMP-12. Proteomic analysis revealed that the sequence is located in the IG domain within the distal tandem IG segment and is homologous only in humans and mice. Even though at least in murine models MMPs and MMP-12 in particular have been implicated in cardiovascular events such as atherogenenesis [37], the only MMP previously described to have a direct effect on titin degradation is MMP-2, which contributes to titin degradation in ischemic and reperfusion-related events [34,37].

We hypothesized that the MMP-12 specific fragment of titin could be potentially useful for monitoring pathologic cardiovascular events and thus as a biomarker. Our hypothesis was that titin degradation fragments may be released and found in the circulation, in a similar way as adjacent proteins in the myocyte, such as troponin, are released and measured, and that the level of titin fragments may be associated to the degree of myocyte damage. Examples of cardiac markers based on troponin, a protein which is in close proximity to and interacts with titin, indicate that release of myocyte-related protein remodeling fragments into the circulation can provide accurate markers of pathology-related remodeling. We investigated this hypothesis by developing a monoclonal antibody and a serum-based assay for the identification of the titin fragment degenerated specifically by MMP-12. We used the assay in three well- characterised populations with different degrees of heart disease, but with comparable gender- and age-composition. The samples were collected by the same hospital staff using standard procedures, hence adding low biological variance and increased clinical significance.

## Methods

### Reagents

All reagents used for experiments were standard high-quality chemicals from Merck (Whitehouse Station, NJ, USA) and Sigma Aldrich (St. Louis, MO, USA). The synthetic peptides used for monoclonal antibody production were purchased from the Chinese Peptide Company, Beijing, China.

### Selection of the peptide for immunization

The amino acid sequence selected for the assay was chosen from mass spectrometry performed on human tissue [36]. Peptide fragments were identified using the Uniprot database, with the accession number C0JYZ2. The sequence NVTVEARLIK located between amino acid position 12670’ and 12679’ (titin) was selected as the immunogen. The first 10 amino acids of each free end of the sequences identified were regarded as a target sequence. All relevant sequences were analyzed for homology and then blasted for homology using the NPS@: network protein sequence analysis [38]. The sequence was identified by Uniprot and PBIL (Pole Bio Informatique Lyonnais) network protein sequence analysis in UNIPROT-SWISSPROT databases and was found to be unique to human and mouse titin. The full 10- amino acid sequence was also blasted with 1 and 2 mismatched amino acids as well as with 95%, 90%, 85%, 80%, 75%, 70% similarity levels. In all cases the 10 amino acid sequence was found to be unique for human and mouse titin. The selected sequence was also found to be present in 6 out of 8 titin isoforms produced by alternative splicing. These were isoforms 3 (small cardiac N2B), 7 (cardiac novex-2) and 8 (cardiac novex-1) that are known to be present in cardiac muscle (Uniprot accession numbers Q8WZ42-3, Q8WZ42-7 and Q8WZ42-8 respectively) and isoforms 2, 4 and 5 (Uniprot accession numbers Q8WZ42-2, Q8WZ42-4 and Q8WZ42-5 respectively) [39].

The 6 amino acids from the C terminal end of the selected peptide (12675’ EARLIK 12679’) were also blasted using NPS to assess and identify the sequence similarity with potential cross-reactive sequences found in the circulation. This only revealed a similarity with a sequence found in sterile, alpha motif domain-containing protein, while the remaining hits were found to be titin-specific.

### Immunization procedure

Six 4–6 week old Balb/C mice were immunized subcutaneously in the abdomen with 200μL emulsified antigen (50 μg per immunization), using Freund’s incomplete adjuvant (KLH-NVTVEARLIK). Immunizations were performed at two-week intervals until stable titre levels were obtained. At each bleeding, the serum antibody titre was measured and the mice with the highest antibody titre and best reactivity towards serum and urine were selected for fusion. The selected mice were boosted intravenously with 50 μg immunogen in 100μL 0.9% sodium chloride solution three days before surgical removal of the spleen for cell fusion. The study was approved by the Beijing laboratory animal administration office under approval number 200911250.

### Fusion and antibody screening

The fusion procedure has been described elsewhere [40]. Briefly, mouse spleen cells were fused with SP2/0 myeloma fusion partner cells. The hybridoma cells were cloned using a limiting dilution method and transferred into 96-well microtiter plates for further growth. Standard limiting dilution was used to promote monoclonal growth. Supernatants were screened using an indirect ELISA, while the biotinylated peptide Biotin-NVTVEARLIK was used as a catcher peptide on streptavidin-coated microtitre plates.

### Characterization of clones

Native reactivity and peptide-binding of the monoclonal antibodies in human serum was evaluated using a preliminary ELISA with a 10 ng/mL biotinylated peptide coater on a streptavidin-coated microtitre plate and the supernatant from the growing monoclonal hybridoma. Clone specificity was tested against a free synthetic peptide (NVTVEARLIK) and a non-sense synthetic peptide sequence. Isotyping of the monoclonal antibodies was performed using the Clonotyping System-HRP kit, cat.5300-05 (Southern Biotech, Birmingham, AL, USA). The selected clones were purified using Protein G columns according to the manufacturer’s instructions and dialysed (GE Healthcare Life Science, Little Chalfont, Buckinghamshire, UK).

### MMP-12 titin assay protocol

The following competitive ELISA protocol was optimised for use with the MMP-12 titin monoclonal antibody. The selected monoclonal antibodies were labelled with horseradish peroxidase (HRP) using the Lightning-Link Horseradish Peroxidase (HRP) antibody labelling kit according to the manufacturer’s instructions (Innovabioscience, Babraham, Cambridge, UK). A 96-well streptavidin plate (Roche Diagnostics, Basel, Switzerland) was coated with 1.3 ng of the biotinylated synthetic peptide, Biotin-CGG-NVTVEARLIK, dissolved in assay buffer 50 mM Tris BTB (p.H 8 at 20°C) and incubated for 30 minutes at 20°C. 20μL of the peptide calibrator or sample were added to appropriate wells, followed by 100 μL of 40 ng/ml conjugated monoclonal antibody, and incubated for 1 hour at 4°C. Finally, 100 μL tetramethyl benzinidine (TMB) (Kem-En-Tec cat.438OH, Taastrup, Denmark) was added, and the plate was incubated for 15 minutes at 20°C in the dark. All of the above incubation steps included shaking at 300 rpm. After each incubation step the plate was washed five times in washing buffer (20 mM Tris, 50 mM NaCl, pH 7.2). The TMB reaction was stopped by adding 100- μL of stopping solution (1% HCl) and measured at 450 nm with 650 nm as the reference. A calibration curve was plotted using a 4-parametric mathematical fit model with a starting concentration of 1000 ng for the standard peptide following a 2-fold dilution.

### Technical evaluation

From 2-fold dilutions of human serum, linearity was calculated as a percentage of recovery of the 100% sample. The lower limit of detection (LLD) was determined from 21 zero samples (i.e. buffer) and calculated as the mean + 3x standard deviations. The inter- and intra-assay variation was determined by 10 independent runs of six quality control (QC) human serum and mouse heparin plasma samples run in duplicate.

### Tissue specificity

Cardiac and skeletal muscle where collected from an adult mouse and frozen in liquid nitrogen. Mouse tissue was selected for the specificity test because of the 100% homology of the selected antibody sequence between human and mouse, and because of the lack of available human tissue samples for both cardiac and corresponding skeletal tissue. Titin was extracted according to the method described by Granzier and Irving [6]. Briefly, the tissues were pulverized to a fine powder using a mortar and a pestle bathed in liquid nitrogen, and then rapidly added to 9 volumes of hot solubilization buffer (50 mM Tris–HCl, 2% SDS, 10% glycerol, 80 mM DTT) and homogenized for 90 seconds in a 90-95°C water bath. The extracted samples were then immediately cooled down and stored at −20°C.

A buffer exchange was performed using Vivaspin-2 3000 MWCO PES columns (Sartorius Stedim Biotech, Goettingen, Germany) against PBS 1X and the protein concentration was measured using the NanoDrop ND-1000 spectrophotometer (Thermo Scientific, Waltham, Massachusetts, USA). MMP-12 derived titin fragments were measured in the extracted samples using the ELISA assay described above.

### Patients and sample collection

#### Patients

As described in detail below, our study is a case–control study, in which we selected and grouped individuals on the basis of prior knowledge about the presence or absence of well-defined manifestations of ischemic heart disease. The groups were chosen for the sole purpose of investigating connections between biomarkers and differences in the degree or type of ischemic heart disease. Individuals from larger ongoing studies were selected to ensure this analysis compared populations with a similar gender- and age- distribution. Importantly, pre-analytical conditions were similar in all groups.

Ninety individuals with different degrees of atherosclerotic heart disease, were selected from larger studies (DANRISK and DEFAMI), which were actively enrolling patients at Odense University Hospital in Denmark during the same time period. The individuals were divided into three groups of 30. One group consisted of subjects without previous CVD and no coronary calcium detectable on a CT scan (CT-noCa); another 30 asymptomatic subjects from the DANRISK study had no previous CVD but were diagnosed with subclinical CVD due to their severe subclinical coronary calcium shown on CT scans (CT-plusCa); and the remaining 30 patients had acute AMI, and were from the DEFAMI study (Table 1). All samples from these individuals were pre-analytically handled and stored by the analytical unit at Odense University Hospital.

**Table 1:** Demographics of each group in the study, mean values and standard deviation

	**CT-noCa**	**CT-plusCa**	**AMI**	**ANOVA**
	**n = 30**	**n = 30**	**n = 30**	**p-value**
**Age (yrs.)**	60.2	60.3	64.5**	0.001
	[59.8; 60.7]	[60.0; 60.6]	[56.0; 73.0]	
**Triglycerides**^**A**^**(mmol/L)**	1.57	1.49	1.30	0.52
	[0.85; 2.93]	[1.00; 2.21]	[0.78; 2.19]	
**HDL Cholesterol**^**A**^**(mmol/L)**	1.28	1.36	1.13	0.07
	[0.93; 1.75]	[0.98; 1.89]	[0.83; 1.52]	
**LDL Cholesterol (mmol/L)**	3.14	3.26	2.93	0.47
	[2.13; 4.16]	[2.29; 4.22]	[1.74; 4.11]	
**Total Cholesterol (mmol/L)**	5.25	5.47	4.74	0.07
	[4.06; 6.43]	[4.29; 6.64]	[3.44; 6.04]	
**Systolic Blood Pressure (mm Hg)**	145	147	159*	0.03
	[131; 159]	[128; 167]	[132; 187]	
**Diastolic Blood Pressure (mm Hg)**	86	85	88	0.80
	[76; 95]	[75; 96]	[72; 103]	
**Agatston score**	-	1299	-	-
		[717; 2352]		
**Heartscore**^**A**^	5.29	6.38	8.00	0.07
	[2.89; 9.68]	[2.96; 13.7]	[4.01; 16.0]	
**Osteopontin**^**A**^	83	99	111	0.08
	[52; 132]	[67; 145]	[61; 201]	
**Osteoprotegerin**^**A**^	1583	1704	2170**	0.0007
	[1144; 2189]	[1335; 2175]	[1487; 3168]	
**Fibulin**^**A**^	63	63	78**	0.0006
	[49; 80]	[50; 80]	[62; 99]	

CT-noCa: Asymptomatic individuals without detectable coronary calcium; CT-plusCa: Asymptomatic CVD with high coronary calcium; AMI (Acute Myocardial Infarction): patients with NSTEMI-ACS. Mean values [+/− STD)], ^A^ Geometric mean values [+/− STD)], and p-value from one-way of analysis of variance (ANOVA). The level of significance of p-values from comparison of each group against the control group ‘CT-noCa‘was adjusted for multiple comparisons by the method of Dunnet (*: p < 0.05; **: p < 0.01; ***: p < 0.001).

In the DANRISK-study [41], a random sample of 1825 middle–aged men and women was in 2009 invited for screening for coronary disease. Screening included a cardiac CT scan, followed by an estimation of the amount of coronary calcium using the Agatston score. Of the invited subjects, 1257 were considered for inclusion in the study. From this group were excluded those with prior ischemic heart disease or diabetes mellitus (n = 100), leaving a total of 1157 patients for analysis. Among the individuals aged 60 years (n = 647), we selected 30 patients without coronary calcium (CT-noCa) and another 30 with an Agatston score ≥400 (CT-plusCa).

In the DEFAMI-study, all patients admitted between October 1 2009 and April 30 2010 at any clinical department at Odense University Hospital and having troponin analysis performed because of suspected acute coronary syndrome were enrolled. Blood sampling was done as part of a large Biobank within the first 24 hours of symptom onset. Of the 822 patients in the DEFAMI study, 30 individuals with non-ST elevation myocardial infarction (NSTEMI, n = 24) or unstable angina (n = 6) were selected to participate in the current study. Their age was approximately 60 years and their gender distribution matched the two above-mentioned DANRISK subgroups. NSTEMI was defined as increased TnI levels above 0.03 μg/l, but ECG without ST-elevations. Unstable angina was defined as chest pain occurring during rest or minimal physical exertion. The mean TnI level in the NSTEMI-ACS group was 0.62 μg/l (range 0.01-5.23 μg/l). The NSTEMI-ACS patient group was chosen because in these patients it was possible to obtain blood samples before medication with heparin or other interventions for acute myocardial infarction, which could interfere with biochemical assays.

In these three patient groups, hypertension was defined as the use of antihypertensive medical treatment and diabetes as the use of anti-diabetic medication. The patients were considered to have hypercholesterolemia if the diagnosis was stated in the patient file or if the patient was taking cholesterol-lowering treatment.

Systolic and diastolic blood pressure was measured on the same day as blood sampling. Agatston score was calculated in the two groups of patients undergoing cardiac CT. Troponin in the NSTEMI-ACS group was measured prior to the blood sampling. Total cholesterol, LDL, HDL and triglycerides were measured prior to the blood sampling. Blood samples were drawn in tubes with EDTA and centrifuged at 200 g for 10 min. Plasma was stored at −80°C until biochemical analysis.

Protocols for all three studies at Odense University Hospital were approved by the Regional Ethics Committee before the initiation of the trials. Informed consent was obtained from all participating patients.

### Statistical analysis

The data of HDL cholesterol, heartscore, the ratio between LDL and HDL cholesterol, and levels of HDL triglycerides, osteoprotegrin (OPG), fibulin, osteopontin (OPN) and titin were logarithmically transformed to obtain symmetry of variance. One-way analysis of variance (ANOVA) was used for comparison among groups. If ANOVA revealed a statistically significant difference between groups, each of the groups (CT-plusCa and AMI) was compared with the control group (CT-noCa) with the level of significance adjusted for multiple comparisons by the method of Dunnet. For each group of CT-plusCa and AMI, the markers showing a statistically significant difference between groups in the ANOVA were further investigated by ROC curve against the CT-noCa group.

The relationship between titin and other factors (triglycerides, LDL-cholesterol, HDL-cholesterol, total cholesterol, systolic blood pressure, diastolic blood pressure, and age) was investigated by univariate linear regression analysis and multivariate analysis of variance using the general linear model procedure. The diagnostic value of the assay was calculated by receiver-operating characteristic (ROC) curve plots.

The Statistical Analysis System (SAS Institute, Cary, NC, USA) was used for the analyses. The level chosen to indicate statistical significance was 5%.

## Results

### Demographics

Key demographic information and biochemical measurements are presented in Table 1.

### Clone characterization & tissue specificity

**Figure 1:**
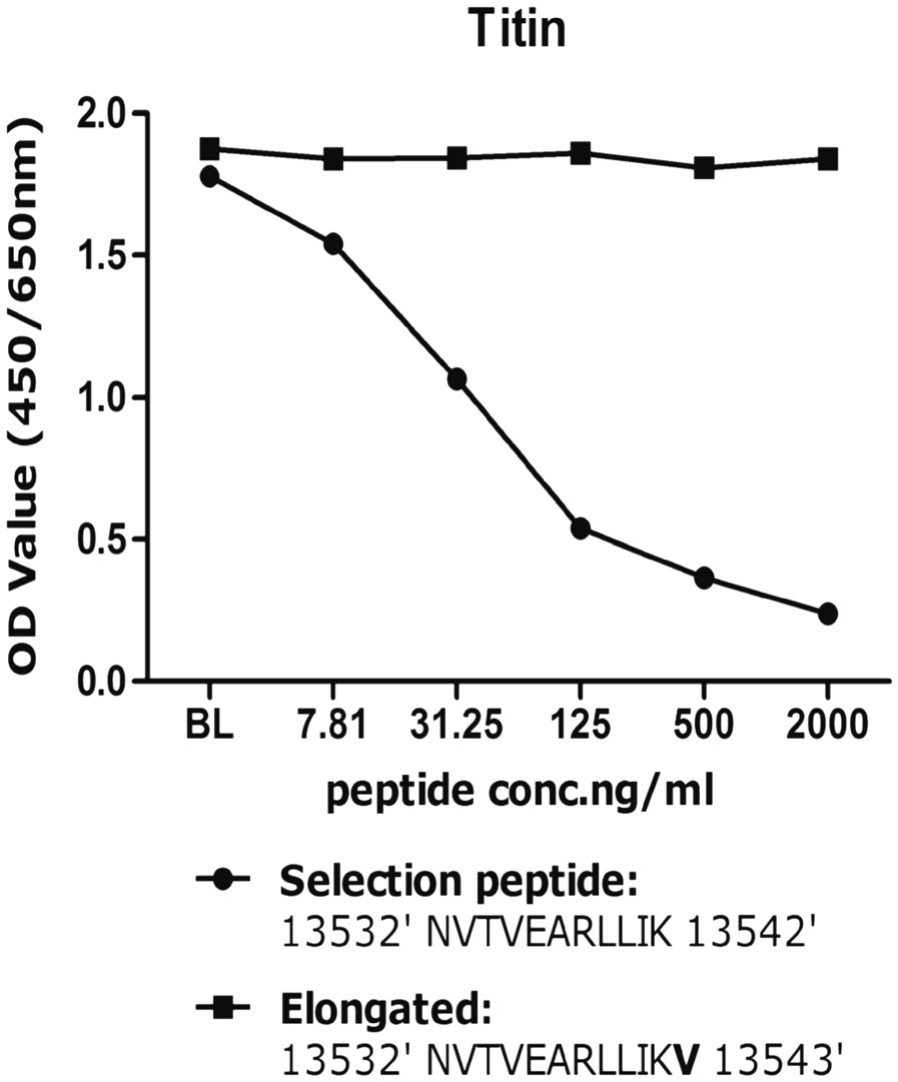
**Specificity of the antibody to the selection peptide vs. non-reactivity to the elongated peptide.** (**A**) The signal was assessed as the optical density at 450 nm, subtracting the background at 650 nm, as a function of peptide concentration. LLD was found to be 5.43. Point zero represents increased signal inhibition and therefore elevated levels of the epitope in serum.

The clone selected for ELISA development was determined to be the IgG1 subtype. The native reaction of this clone was high against human serum. The clone was found to react specifically to the target sequence and not the elongated peptide (Figure 1). The clone showed higher signal inhibition towards mouse cardiac muscle (51% inhibition) than to skeletal mouse extracts (21% inhibition).

### Technical evaluation

The typical standard curve is presented in Figure 1, showing a 4-parametric fit for the assay. The lower limit of detection for the assay was 9.78 ng/mL. Dilution recovery was found to be within 100 ± 15%. The inter- and intra-assay variation was found to be 4.33 and 5.49% respectively.

### Clinical cohort

**Figure 2:**
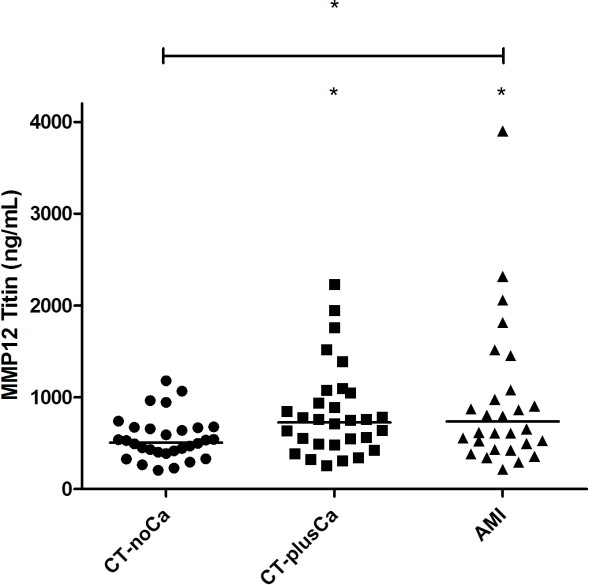
**MMP-12 generated fragments of titin measured in a clinical cohort consisting of individuals with no diagnosed cardiac pathology and no coronary calcium.** (CT-noCa) (n = 30), with high coronary calcium (CT-plusCa, n = 30), acute myocardial infarction (AMI, n = 30). A statistically significant difference between the groups was measured by one- way ANOVA (P < 0.05). The bar shows geometric mean value.

A statistically significant increase in the marker was measured in CT-plusCa and AMI patient groups (Table 2). The geometric mean level of the MMP-12 degraded titin fragment in the CT-noCa group was 506.5 ng/ml. In the AMI patient group, the mean value was 792 ng/ml (56.3% increase, P < 0.05), and in the CT-plusCa group 763 ng/ml (±90.14) (43% increase, P < 0.05) (Figure 2). Although Levene’s test for homogeneity of variance was non-significant (p = 0.07) there was a tendency to higher variance in the CT-plusCa group (standard deviation 74%); AMI (standard deviation 97%) as compared with the CT-noCa group (standard deviation 54%).

**Table 2:** Comparison of levels of MMP-12-generated fragments of titin in the clinical cohort

	**CT-noCa**	**CT-plusCa**	**AMI**
	**n = 30**	**n = 30**	**n = 30**
Geometric mean value(ng/ml)	506.5	759.7	792.5
SEM	43.88	90.14	149.5
% Increase	Control	43%	56.3%
P Value	Control	0.03	0.02

The highest sensitivity and specificity of the assay was found at the cut-off value of 545 ng/ml. At this value the sensitivity and specificity were 73.3% and 63.3% respectively for the CT-plusCa group, while the positive predictive value (PPV) and negative predictive value (NPV) were 64.7% and 68% respectively. In the AMI group, sensitivity and specificity was 67.7% and 63.3% respectively, while the PPV and NPV were 65.6% and 65%.

### ROC values of the assay

The assay was found to have a highly significant ROC value for all patient groups (Table 3).

**Table 3:** ROC values of the assay measuring MMP-12 generated titin fragments, vs. other known cardiac markers

**CT-noCa**	**Vs. CT-plusCa**	**Vs. AMI**
Systolic Blood Pressure	0.54	0.70 **
Total Cholesterol	0.55	0.61
LDL	0.53	0.55
Heart score	0.57	0.70 **
Osteoprotegerin	0.58	0.73 ***
Fibulin-1	0.49	0.74 ***
Osteopontin	0.64 *	0.64 *
**MMP12-Titin**	**0.69** **	0.65 *

### Relationship between titin and other factors

The marker showed no significant correlation with any other physiological measurements. The relationship between titin and the physiological characteristics of triglycerides, LDL-cholesterol, HDL-cholesterol, total cholesterol, systolic blood pressure, diastolic blood pressure, and age was statistically non-significant both in the separate univariate linear regression analysis and in multivariate analysis of variance (Wilks Lambda criteria: CT-noCa: p = 0.79.; CT-plusCa: p = 0.39; AMI: p = 0.74). The level of titin in the AMI patients also showed no significant correlation with the troponin I levels.

## Discussion

The study relied on a well-characterised cohort in which all samples were collected under the same standard operating procedures. The study presents for the first time an assay for in vivo detection of titin degradation fragments that have been associated with abnormal remodeling and cardiac damage. The assay had a good native reactivity in human serum and was technically robust with acceptable inter- and intra-assay variation, dilution recovery and a low limit of detection. The assay detected statistically significant increased levels of the biomarker in all the pathology-related clinical cohorts when compared with non-diseased individuals. The assay has shown increased and statistically significant ROC values for all cohorts. Unlike all but one of the other known cardiac markers measured in the cohorts, the titin degradation marker showed a statistically significant elevation with high ROC values in the CT-plusCa and AMI groups (Table 3). With the exception of osteopontin, no other marker showed a high ROC and significant p-value in the group of CVD asymptomatic individuals with increased coronary calcium levels (CT-plusCa). We believe that the raised titin levels may be a tissue-specific indication of the underlying remodeling processes occurring in the myocyte prior to injury and during pathology development which could be of additional value for early diagnosis of CVD asymptomatic patients. However, a longitudinal study and measurement of the MMP-12 titin marker at a later time point in the development of CVD could be of great value to determine the validity of this hypothesis.

The statistically significant increase in the marker levels in the AMI group may be related to the increased macrophage levels found following AMI at the injury site. Macrophages are implicated in all components of AMI response, which include inflammation, scar formation and remodeling [42]. As will be discussed later, macrophages are the main source of MMP-12 in adult tissue which participates in inflammatory processes and ECMR. Hence, increased levels of MMP-12 or other macrophage-derived proteases acting on the infarcted myocardium may be responsible for protease-driven myocardial remodeling. The raised MMP-12 levels are thought to increase degradation of titin at the site of injury, with the titin fragments indicating myocardial injury.

The marker was found not to be correlated with age, LDL, HDL, total cholesterol, triglycerides, BT systolic or diastolic pressure levels which implies that the titin fragment levels are independent of these markers. The lack of correlation may be also attributed to the fact that this assay measures an MMP-degraded fragment of titin and not total protein, as do tests for the above-mentioned biochemical measurements. We believe that the fact that the marker does not directly correlate with the above measurements is not alarming, because the specific titin marker does not measure total protein levels as the above measurements but rather the increased titin degradation levels which results from chronic abnormal remodeling. Further validation of this finding in additional, larger clinical cohorts, could potentially validate this titin marker’s utilisation in clinical prediction models as an additional independent risk factor to diagnose asymptomatic patients. The marker should be further evaluated for all criteria set for novel cardiovascular risk factors which include its reliability, sensitivity, availability/practicality, clinical significance and value of independent information on prognosis [43,44].

To date there is no adequate relevant literature regarding the specific activity of MMP-12 – a human macrophage elastase - and its effect on cardiac remodeling during either physiology or pathology. Human and animal studies have established the presence and activity of MMPs such as −2, -9 and −7 on cardiac-related remodeling [37,45-52]. Whether the acknowledged MMP-driven activity is dependent on tissue inhibitors of metalloproteinases (TIMPs) or not remains unclear [53]. Even though there is evidence of MMP-12 activity in cardiovascular pathologies such as aortic aneurysms and atherosclerosis [54-58], to our knowledge there is no pre-existing evidence directly implicating MMP-12 activity in cardiac remodeling. The main MMP-12 source in adult tissue is macrophages which have been reported to be an active participant in inflammatory processes and vascular extracellular matrix remodeling [59]. The role of MMP-12 in cardiac pathology is not well understood. Benoit *et al.* showed that MMP-12 expression was increased in cardiac valves taken from patients suffering from infective endocarditis [60].

Biochemical markers consisting of protein fragments from matrix remodeling degradation may be informative of disease pathology and progression, which in turn may be useful for diagnostic and prognostic purposes. These markers could potentially detect changes resulting from intervention strategies and serve as surrogate markers of drug efficacy [61]. The continuous search and utilisation of cardiac specific markers such as cTnI & cTnT, that can provide additional information, could be of real clinical value for improved patient care by aiding an early diagnosis, prognosis risk stratification, therapy selection and close monitoring of patients [29]. The described fragment of titin should be further evaluated in additional clinical cohorts in a longitudinal study in which other cardiac markers such as cTnI & cTnT will also be measured in order to facilitate its categorisation according to the BIPED criteria [22].

The main limitations of this study include the absence of direct quantitative measurements of MMP-12 and other relevant protease levels in the tissue from the clinical cohorts and thus a correlation between the levels of the titin fragment and MMP-12 activity. It should also be noted that we selected and grouped individuals on the basis of knowledge about the presence or absence of well-defined manifestations of ischemic heart disease and to match the groups for age- and gender distributions. However, it is important to note that each group does not necessary reflect all patients with this disease, for example the AMI group, which only included non-STEMI patients. Additionally, the study lacked a detailed assessment of the troponin C and I levels in all patients. Due to their position in the myocyte and their vicinity to the titin location, troponin C and I could have been tested for their correlation to titin. A further limitation is that even though the described fragment is derived from MMP-12 activity in vitro, other proteases or a combination of proteases could potentially generate this fragment in vivo. The fragment’s specificity for cardiac tissue was evaluated in mouse tissue, due to the sequence homology between human and mouse, but in future an assessment in human tissue could provide more clinically reliable information. Due to the presence of titin in both skeletal and cardiac muscle, measurement of the marker in other pathologies such as muscular dystrophies could indicate a broader use for this marker. The study also lacks immunohistochemistry and western blot data due to the lack of tissue from the enrolled patients. Despite numerous attempts to obtain additional western blot data using mouse tissue instead, the clone currently used could not be utilised for western blot use. Additional clones are being raised by new immunisations that could produce a clone that can be used for western blotting and immunohistochemistry. Even though the marker can be accurately measured in serum, it may be also present in other body fluids such as urine and saliva. These have not been assessed in this study due to lack of relevant material. However, when the material is made available it will be assessed accordingly. This will allow for additional assessment of the marker and the influence of renal function on its presence and levels. Measurement of the marker in a larger cohort and with additional CVD related pathologies will also allow for a better description of the marker. Measurement of additional biomarkers such as troponins and CKMB and direct comparisons of these with MMP-12 generated titin fragments will add to our understanding of the marker and its potential utility. Finally, even though the marker was found to be elevated in the tested clinical groups, the result represents only a “snapshot” in the complex cardiac remodeling process that occurs during pathologic events. A longitudinal study including detailed monitoring of the marker during cardiac remodeling is needed to better describe the marker, together with further assay optimisation.

## Conclusion

We have developed and validated a serum-based competitive assay using a specific monoclonal antibody for the detection of the titin sequence, titin-12670 (NVTVEARLIK). To our knowledge this is the first neoepitope biomarker that specifically detects in serum, in vivo, titin remodeling activity and is related to cardiac damage in the cohorts examined in this study. The data suggest that there is a promising potential for the use of such biomarkers to assesscardiovascular related diseases in a clinical setting. Further testing in additional cohorts is required to validate our findings.

## Abbreviations

MMP: Matrix metalloproteinase; ECM: Extracellular matrix; ECMR: Extracellular matrix remodeling; LLD: Lower limit of detection; CVD: Cardiovascular disease; CABG: Coronary artery bypass graft; HCC: High coronary calcium; AMI: Acute myocardial infarction; ELISA: Enzyme linked immunosorbent assay.

## Competing interests

Efstathios Vassiliadis, Natasha Barascuk, Federica Genovese, Xiaoliang Chen, Qinlong Zheng, Diana J. Leeming and Morten A. Karsdal are full-time employees of Nordic Bioscience.

## Authors’ contributions

EV carried out sequence selection, ELISA development, draft manuscript, statistical analysis, tissue extractions, conceived study and study coordination. LMR provided patient samples, helped with the description of cohorts and the discussion and actively contributed to ideas for the project. IB performed the statistical analysis and added key parts of the methods, results and discussion. DVL developed the ELISA and added to the manuscript. NB carried out immunoassays, tissue extraction and drafted additions to the manuscript. RC has creatively added to the manuscript discussion and introduction. SH has collected patient samples and biochemical measurements and helped in the description of the cohorts. LS has collected patient samples and biochemical measurements and helped in the description of the cohorts. ACPD has collected patient samples and biochemical measurements and helped in the description of the cohorts. FG has performed tissue extractions, immunoassays and sections in both materials and methods and results. KLD has helped with the mass spectrometry selection process and collection of data as well as creative inputs into the manuscript. QZ has performed mice immunisations, clone selection, antibody production and early immunoassay characterisation data. XC has performed mice immunisations, clone selection, antibody production and early immunoassay characterisation data. DJL has creatively added into the manuscript building process and ELISA optimisation steps. CC has creatively added into the formation of manuscript, read and approved the final version. MK has creatively added into the ELISA optimisation steps, formation of manuscript, study coordination, read and approved the final version. All authors read and approved the final manuscript.
